# Exploiting biased reptation for continuous flow preparative DNA fractionation in a versatile microfluidic platform

**DOI:** 10.1038/micronano.2017.1

**Published:** 2017-05-22

**Authors:** Burcu Gumuscu, Johan G. Bomer, Hans L. de Boer, Albert van den Berg, Jan C. T. Eijkel

**Affiliations:** 1 BIOS Lab-on-a-Chip Group, MESA+ Institute for Nanotechnology, MIRA Institute for Biomedical Technology and Technical Medicine, University of Twente, 7500 AE Enschede, The Netherlands

**Keywords:** agarose gel, biased reptation, continuous flow DNA separation, DNA purification, preparative fractionation

## Abstract

A new approach is presented for preparative, continuous flow fractionation of sub-10-kbp DNA fragments, which exploits the variation in the field-dependent mobility of the DNA molecules based on their length. Orthogonally pulsed electric fields of significantly different magnitudes are applied to a microchip filled with a sieving matrix of 1.2% agarose gel. Using this method, we demonstrate a high-resolution separation of 0.5, 1, 2, 5, and 10 kbp DNA fragments within 2 min. During the separation, DNA fragments are also purified from other ionic species. Preparative fractionation of sub-10-kbp DNA molecules plays an important role in second-generation sequencing. The presented device performs rapid high-resolution fractionation and it can be reliably manufactured with simple microfabrication procedures.

## Introduction

Standard gel electrophoresis has been widely utilized for DNA fractionation in various genotyping and sequencing applications^1–3^. This method has the advantages of simplicity, versatility, and reproducibility. However, it suffers from long processing times on the order of a few tens of hours^2,3^. For instance, the Bio-Rad CHEF-DR device performs fractionation of 5–120 kbp DNA using pulsed-field gel electrophoresis (PFGE) in 25 h (Refs. 4,5). Trends toward second-generation sequencing motivate the replacement of standard gel electrophoresis with microchip-based systems, which could provide efficient platforms to minimize the processing time and to perform optimal DNA fractionation^6–8^.

Micro- and nanofabricated post arrays—that resemble a gel matrix with well-ordered and identical pores—have been integrated in microchip-based systems, increasing both the understanding of DNA separation principles and the fractionation’s efficiency and speed. Two decades ago, Volkmuth and Austin^9^ introduced a patterned micro-post array in a microfluidic electrophoresis platform, in which DNA fragments were separated by biased reptation under a DC electric field. Later, Duke *et al.* separated large fragments in a range of 60–135 kbp in a microfabricated array using DNA reorientation—or the ‘switchback’ principle. Although the switchback principle is similar to what is used in PFGE, the device yields much faster separations owing to its sparse and regular sieving array^10^. In a later work, Kaji *et al.* studied a three-dimensional (3D) nanopost array as an optimal separation matrix for DNA fragments over a few kbps under a DC electric field, resulting in similarly fast separations due to the sparse matrix^11^.

With the aim of increasing the sample throughput and further facilitating the sample recovery, a continuous flow separation was developed. Huang *et al.*^12^ performed continuous flow PFGE in a micromachined post array by applying pulsed electric fields of slightly unequal strengths. DNA fragments ranging between 61 and 209 kbp were separated within 15 s in the ‘DNA prism’. A comparable device was later incorporated into a gene analysis system and is currently being marketed by the company Pathogenix, to detect the genome of rare pathogenic bacteria from complex mixtures^13^. The operation time of this device is reported as ~1.5 h to fractionate 60–200 kbp DNA^13,14^. Another reported variant of the ‘DNA prism’ device was a self-patterned 3D crystalline nanoarray used for fractionating smaller DNA molecules (2–50 kbp) in a continuous flow setup^15^. Other separation principles, including Ogston sieving, entropic trapping, and electrostatic sieving, were demonstrated by Fu *et al.* using a nanofabricated two-dimensional (2D) sieving array operated under pulsed electric fields to fractionate DNA in a continuous flow^16^.

The micro- and nanofabricated sieving devices briefly reviewed above allow for the optimization of the separation process by using spatially controlled sieving matrices that optimally exploit the basic physical principles of DNA separation. However, defect-free fabrication of the 3D nanostructures, such as the nanopost array^11^ and the crystalline nanoarray^15^, is extremely challenging. Although the manufacturing of 2D nanostructures (for example, the anisotropic nanofluidic sieving array^16^) is relatively easier, these nanostructures intrinsically yield low sample throughput. An ideal sieving matrix should thus have a simple design and facile fabrication steps yet provide high-resolution and high-throughput separation.

Remarkably, a relatively larger device (on the order of a few cm) filled with bulk agarose gel has been demonstrated for the purification and concentration of high molecular weight DNA (on the order of tens of thousands of bp), using the field-dependent mobility of DNA in a method called synchronous coefficient of drag alteration (SCODA)^17–19^. However, this study overlooked the fact that the field-dependent mobility depends on the DNA fragment size, which will be shown for the first time in this work to provide an alternative method for continuous flow DNA fractionation that has hitherto not been demonstrated.

We demonstrate a microscale gel electrophoresis (μGEL) device operated under orthogonal pulsed electric fields with significantly differing magnitudes for continuous flow fractionation of sub-10-kbp DNA molecules. Preparative fractionation is shown here over a broad frequency range (3 decades). The μGEL device presents three major advances over the aforementioned microchip-based systems. First, agarose gel is used as the sieving matrix. This requires much less effort to fabricate a defect-free 3D network of nanopores for high-resolution separations, and offers considerable flexibility in varying the pore size for the DNA fragments of interest. Second, field-dependent mobility is explained and experimentally shown as a new separation mechanism for DNA fractionation. Third, we can combine both the separation and purification functions by maximally exploiting the field-dependent mobility. Fragments in a range of 0.5–10 kbp are separated from each other and from other ionic species (fluorescein sodium salt was utilized in this work as an example) in a continuous flow.

## Materials and methods

### Microchip fabrication

**Figure 1:**
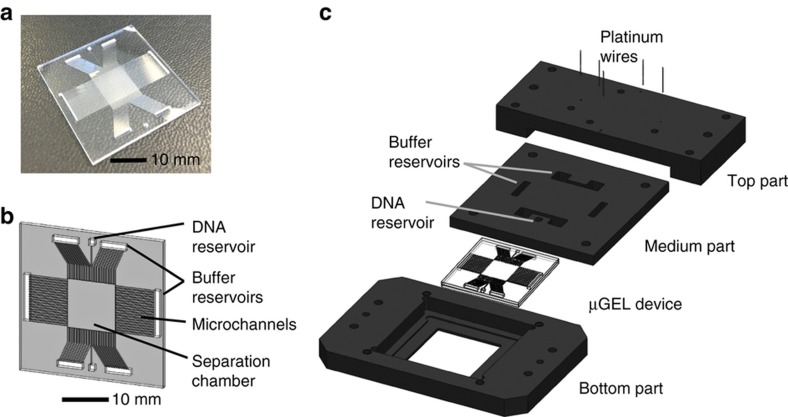
(**a**) An image of the fabricated microchip made of glass. (**b**) Schematic illustration of the microchip layout, including the DNA reservoir, buffer reservoirs, microchannels, and separation chamber. (**c**) Schematic illustration of the in-house made chip holder made of Delrin. The chip holder consists of three main parts. The bottom part has a window that enables the observation of DNA fractionation on an inverted epifluorescence microscope. The middle part has slits serving as the buffer reservoirs in direct contact with the buffer reservoirs on the microchip. The buffer reservoirs in the middle part are filled with buffer to enable contact with the platinum wires. The top part has holes to align the platinum wires to the reservoirs in the middle part. The wires are all connected to the power source.

The microchips were fabricated in the MESA+ cleanroom facility at the University of Twente. Fused silica glass was selected for its high optical clarity and its smooth microchannel surfaces after a reactive ion etching (DRIE) process. Microchips consisted of a processed top and an unprocessed bottom layers. The top layer contained the separation chamber, microchannels, and buffer reservoirs. A bonded microchip is presented in Figures 1a and b, consisting of a 10 by 10 mm square chamber connected to the buffer reservoirs via microchannels (50 μm×10 mm, 50 μm periodicity) on each side. The microchannels serve to generate a uniform electric field over the separation chamber. The overall dimension of the microchip is 35 by 35 mm.

**Figure 2:**
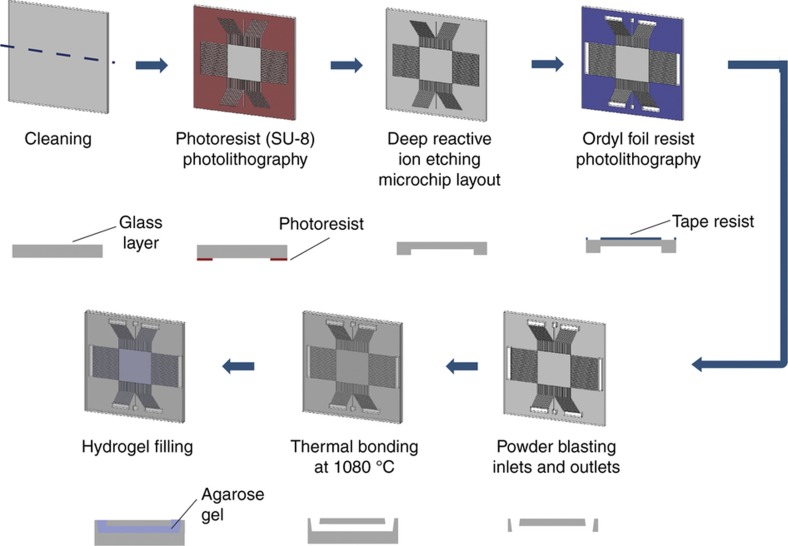
Schematic illustration of the microchip fabrication. The drawings at the bottom of the schematics present the cross sectional view along the blue dashed line in the top-left drawing. After the cleaning step, the layout of the microchip is defined by a photolithography step, which is followed by a reactive ion etching step. After this, the back side of the microchip is coated using Ordyl foil resist. The fluidic access holes are opened by performing photolithography and powder blasting steps, respectively. The processed layer is thermally bonded with an unprocessed layer to form microchannels. The microchip is finally filled with 1.2% agarose gel via capillary action.

DRIE was utilized to define the microchannels and separation chamber and the process flow is shown in Figure 2. Before DRIE, the wafer was spin-coated with SU-8 (MicroChem), which served as a mask for the underlying surface. The SU-8 layer was patterned using photolithography and the residual SU-8 layer was stripped in a piranha bath after the DRIE process. The height of the structures was measured as 20 μm using a Dektak 150 Surface Profiler (Veeco). Buffer reservoirs were opened on the back side of the top layer. After lamination and photolithography, the Ordyl foil resist (BF410, Ordyl) was developed using a NaHCO_3_ (0.2%, w/v, Sigma Aldrich, Germany) solution. Subsequently, a powder blasting process was performed and the Ordyl foil was removed using acetone. Finally, both top and bottom layers were cleaned and thermally bonded at 1080 °C. The bonded wafers were then diced into individual microchips^20^.

Agarose hydrogel was prepared by dissolving agarose powder (1.2%, w/v, Invitrogen) in deionized water. The agarose gel with a 1.2% concentration was chosen because it yields the highest resolution separation for 0.5–10 kbp DNA fragments in the microchip when compared to the agarose gels with 0.5, 1, and 1.5% concentrations. The mixture was boiled in a microwave and pipetted into the microchip that was warmed to 70 °C on a hot plate. The empty space in the microchip was filled entirely by capillary forces^20,21^. The microchip was then immersed in a buffer solution overnight. The agarose gel expanded during the immersion process due to swelling, which ensured an airtight filling of the microchannels and eliminated leakage and air bubbles. The buffer solution consisted of tris borate EDTA (TBE, 0.1×, Invitrogen), β-mercaptoethanol (3%, w/v, Sigma Aldrich), and deionized water.

In our preliminary experiments, we used 0.5, 1, 1.2, and 1.5% agarose concentrations to perform the separation. Among those experiments, we obtained the best resolution using the 1.2% agarose gel for the 0.5–10 kbp fragments.

### Sample preparation

Individual DNA fragments (0.5, 1, 2, 5, and 10 kbp) were obtained from New England BioLabs. Fragments were labeled with an intercalating fluorescent dye YOYO-1 (Invitrogen) at a 1:5 dye-to-bp ratio and the final DNA concentration was 12.5 ng μL^−1^. YOYO-1 solution was added before the DNA solution to prevent precipitation. The mixture was incubated at room temperature for 1 h, and then 0.1% (v/v) β-mercaptoethanol was added to prevent photo bleaching. The final mixture was stable at room temperature.

### Experiment setup

An epifluorescence microscope (Leica, DM-IRM, Germany) equipped with a thermoelectrically cooled CCD camera (Hamamatsu ORCA-ER C4742-80-12AG) and GFP filter cube (Chroma) was used for fluorescence imaging. A 100 W mercury lamp (Leica) was set to the highest intensity level for illumination. Electric fields were applied to the microchip using a LabSmith HVS448LC High Voltage Sequencer (USA). In-house fabricated platinum electrode connectors were mounted in an in-house fabricated chip holder to apply the electric fields. Figure 1c illustrates the chip holder and connections.

### Simulations

**Figure 3:**
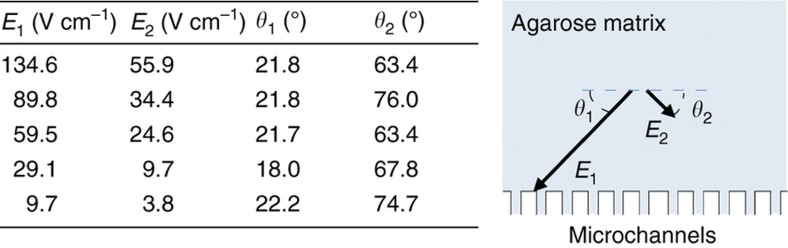
Magnitude of electric field strengths (left) and angles *θ* of the applied electric fields relative to the *x* axis (right).

Two-dimensional finite element calculations of the μGEL device were performed to estimate the electric field distribution in the separation chamber, using the AC/DC module in the COMSOL Multiphysics 5.2 software in stationary conditions. The agarose gel and TBE buffer-filled separation chamber was modeled with a specific conductivity of 4.20 S m^2^ and a relative permittivity of 80. The potentials applied to the reservoirs were defined as shown in Figure 3. The system responses to the applied potentials were governed by Maxwell’s equations under the electric currents interface in the software.

### Resolution calculations

We calculated the separation resolution between two adjacent flow streams *R*_s_=Δ*X*/(2*σ*_1_+2*σ*_2_), where Δ*X* is the spatial distance between the streams, and *σ*_1_ and *σ*_2_ are the standard deviations (s.d.) of the stream widths.

### Image processing

Matlab software was used to plot the fluorescence intensity graphs. To obtain images with comparable intensities, the background of the images was subtracted, the noise was reduced by applying a Gaussian filter to every 5×5 group of pixels, and the contrast was increased by 2%. The fluorescence intensity profile was plotted by drawing a line along the *y* axis of the image (in front of the microchannels), where all the samples were collected. The resulting intensity plots were used to calculate the resolution of the separation. Resolution values between peaks are provided in Table 1.

## Results and discussion

**Figure 4:**
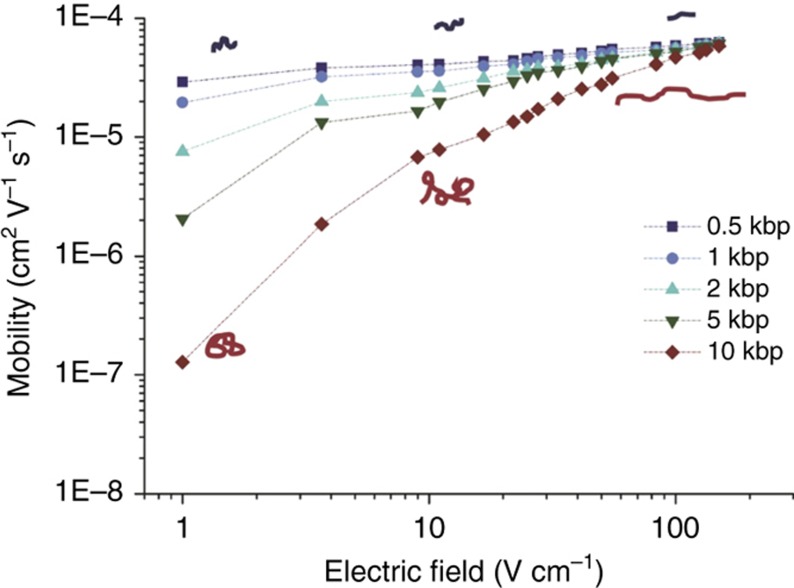
Measured mobility of individual DNA fragments as a function of the electric field. The red and dark blue figures illustrate the molecular conformation of large and small fragments at low and high electric fields, respectively.

Due to the negative charge on their phosphate backbone, DNA molecules move against an applied electrical field as a result of Coulomb forces. DNA strands of different lengths can be separated by applying an electrical field in a porous gel matrix. The DNA velocity is thereby determined by both the electrical force and the size-dependent friction with the gel matrix. Three physical phenomena are crucial to understand the separation process described in this paper: the size dependence of the DNA mobility in the gel matrix, the field-strength dependence of the DNA mobility, and the time needed for the reorientation of the DNA molecules upon changes in the field direction (also called as ‘switchback’ mechanism). The size dependence of the mobility is caused by the tendency of DNA molecules to coil around the gel strands, which increases their friction. This tendency and increased friction increases with the DNA length. The field-strength dependence of the mobility stems from the tendency of the DNA molecules to align themselves in the field direction, which increases with increasing field strength. As a result, when the electric field direction is changed, the reorientation time depends on the DNA mobility and length. Figure 4 demonstrates both the size- and field-dependency of the mobility for different DNA lengths measured in the μGEL device. It will be demonstrated that by a combination of these properties, the μGEL device can separate DNA molecules of different lengths in continuous flow with a high resolution. This separation can be achieved by alternately applying high and low electric field strengths in orthogonal directions.

Figure 2 outlines the microchip fabrication and hydrogel patterning processes, while Figure 1 presents the design of the μGEL device and the chip holder used in the experiments (see MATERIALS AND METHODS). The microchip consists of a 10 mm by 10 mm chamber connected to buffer reservoirs via parallel microchannel arrays (50 μm×10 mm, 50 μm periodicity) on four sides. The microchannels provide a high-resistance area, preventing current leakage from the separation chamber and generating a fairly uniform electric field (Supplementary Figure S1)^20,22^.

In all separation experiments, two electric fields, *E*_1_ and *E*_2_, were alternately applied across the separation matrix at various frequencies. Figure 3 presents the magnitude and angle at the injection point—relative to the *x* axis—for all the transverse electric fields. Throughout the experiments, the electric field-strength ratio *E*_1_*/E*_2_ at the injection point varied between 2.4 and 3, while the angle between the fields varied around ~90° (85–98°). The DNA fragments were continuously injected into the agarose matrix, and separated fragments were continuously collected at different sides of the microchannels.

Supplementary Figure S1 shows the simulated electric field distribution in the separation matrix at typical *E*_1_ and *E*_2_ values. Both the magnitude and direction of the electric fields vary slightly over the μGEL device. On approach of the collection point, the angle between *E*_1_ and *E*_2_ becomes more acute (up to 60° at some locations in the microchip) while the ratio *E*_1_*/E*_2_ gradually increases (up to 3.1) as calculated using the COMSOL Multiphysics software.

**Figure 5:**
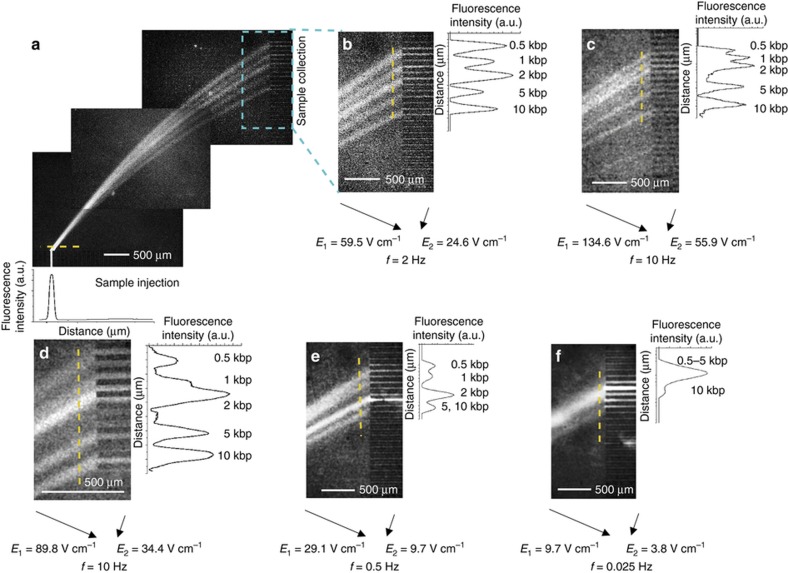
DNA fractionation in a continuous flow. (**a**–**f**) Fluorescence images of the fractionating 0.5–10 kbp DNA in an agarose sieving matrix. The intensity profiles (right) were obtained from the fluorescent images (left) by scanning over the yellow dashed line. Each image was recorded with a 12 s exposure time.

**Figure 6:**
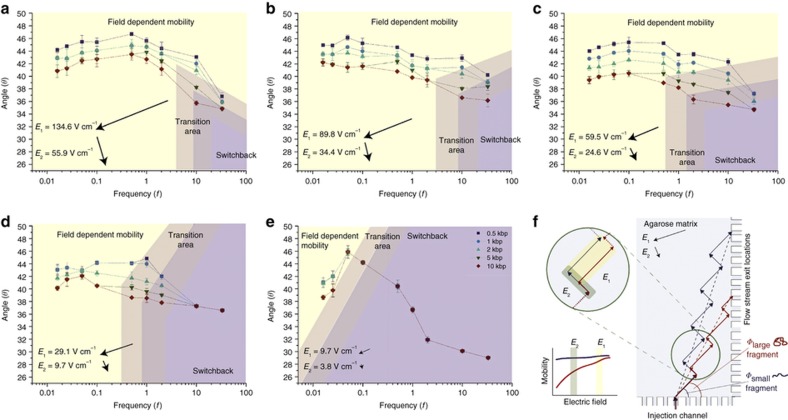
(**a**–**e**) Frequency spectra of the deflection angle *θ* for various DNA fragments under different field strengths. Error bars show s.d. (*n*=2). (**f**) Schematic representation of the separation principle at high electric field strength and low frequency. Blue and red dashed lines present the net migration trajectory of each fragment. *Φ* is the deflection angle of the DNA fragments.

We investigated the continuous flow separations of 0.5, 1, 2, 5, and 10 kbp fragments, at a wide range of the DC electric fields *E*_1_ and *E*_2_ from 1 to 135 V cm^−1^ and switching frequencies from 0.016 to 33 Hz. Figure 5 shows photomicrographs of the flow streams of the individual DNA fragments observed at the various electric fields and switching frequencies. The deflection angle (*Φ*) between the initial stream and the flow stream exit locations was measured to quantify the separation between the fragments (Figure 6f).

The semi-log plots of the deflection angle as a function of the switching frequency, given in Figures 6a–e, show that the DNA fragments can be separated over more than three decades of frequency. To explain this observed behavior, we propose a mechanism based on a combination of the field-dependent mobility and the ‘switchback’ mechanisms, with the former mechanism dominating at low frequencies and the latter mechanism dominating at higher frequencies. The switchback mechanism was previously demonstrated in micromachined devices by Huang *et al.*^12^ and Zeng *et al.*^15^, and theoretically analyzed by Chen *et al.*^23^. When the applied field switches direction, the DNA fragments need time to reorient, $t_{\mathrm{or}}\approx \frac{L}{µE}$, where *L* is the DNA contour length, *μ* the DNA mobility, and *E* is the applied field^19^. This reorientation time is shorter in the large field *E*_1_ than in the small field *E*_2_. The corresponding reorientation frequencies are $f_{\mathrm{or},1}=\frac{1}{2t_{\mathrm{or},1}}\approx \frac{µE_{1}}{2L_{\mathrm{DNA}}}$and $f_{\mathrm{or},2}=\frac{1}{2t_{\mathrm{or},2}}\approx \frac{µE_{2}}{2L_{\mathrm{DNA}}}$. At a switching frequency below $f_{\mathrm{or},1}$and above $f_{\mathrm{or},2}$, DNA molecules can only reorient in the strong field (*E*_1_) and will follow the field vector of this field. At frequencies below $f_{\mathrm{or},2}$ or above $f_{\mathrm{or},1}$ DNA will follow an intermediate trajectory. As the reorientation frequencies are also a function of the DNA length, the migration angle at frequencies $\frac{µE_{2}}{2L_{\mathrm{DNA}}}<f<\frac{µE_{1}}{2L_{\mathrm{DNA}}}$ depends on the DNA length *L*_DNA_, enabling separation around these frequencies. In our system the fractionation at these frequencies can be partially explained by the switchback mechanism. In Figures 6a–e, the frequencies around and above these values are indicated with a purple background.

The physics of DNA reorientation in agarose gels was studied by Aakerman *et al.*^24^ The reorientation frequencies of DNA fragments in the μGEL device lie between 2.4 Hz for 10 kbp fragments and 50 Hz for 0.5 kbp fragments at an applied field of 59.5 V cm^−1^. Optical measurements in the μGEL device confirmed the validity of the equation $f_{\mathrm{or}}=\frac{1}{2t_{\mathrm{or}}}\approx \frac{µE}{2L_{\mathrm{DNA}}}$ for the switching frequency (Supplementary Figure S3). Calculated orientation times for the 10-kbp fragments were 0.24 s when *E*_1_ was switched to *E*_2_, and 0.42 s when *E*_2_ was switched to *E*_1_, when the magnitudes were 59.5 V cm^−1^ (*E*_1_) and 24.6 V cm^−1^ (*E*_2_). The observed reorientation times were found to be in good agreement with theory, calculated as 0.2 and 0.4 s, respectively. At frequencies far below $f_{\mathrm{or},2}=\frac{1}{2t_{\mathrm{or},2}}\approx \frac{µE_{2}}{2L_{\mathrm{DNA}}}$, the DNA fragments will quickly reorient along the new electric field direction and will spend most of the application time moving in steady state along the field^20^. Different from the predictions of the switchback mechanism, we still observe separation at these frequencies. The fractionation observed at these frequencies can now be explained by the variation in the field-dependent mobility of the DNA molecules based on their length. The biased reptation with the orientation principle states that the mobility, *μ*, of a DNA fragment in an agarose sieving matrix is a function of the electric field and DNA contour length^23,25–28^. This behavior was experimentally confirmed in the μGEL device. Figure 4 shows the measured field-dependent mobilities. At low frequencies, the reorientation time can be neglected and the migration trajectories can simply be calculated by adding the trajectories at the high (*E*_1_) and low (*E*_2_) fields (Figure 6f). The larger the DNA fragment, the larger the mobility increases between the two applied fields Δ*μ*=d*μ*/dE*(*E*_1_−*E*_2_) (Figures 4 and 6f), leading to fragments of different sizes following different trajectories.

Adding the contributions of both separation mechanisms, the migration angle of the DNA molecules with respect to the horizontal axis, *Φ*, can be approximated by the following equation:

(1)Φ=atan(sinθ1+(E2µ2−2fLE1µ1−2fL)sinθ2cosθ1−(E2µ2−2fLE1µ1−2fL)cosθ2)

Here *μ*_1,2_ is the mobility of the fragments at the two applied fields *E*_1,2_; *θ*_1,2_ is the angle between the transverse electric fields, *E*_1_ or *E*_2_, and the *x* axis; *f* the applied frequency; and *L* is the DNA length.

The calculated (Equation (1)) and measured deflection angles of the fragments for the 59.5 V cm^−1^ (*E*_1_) and 22.4 V cm^−1^ (*E*_2_) fields are presented in the Supplementary Figure S2. Comparing S2a and S2b shows that four flow streams are obtained at all the frequencies below 1 Hz (Figures 5a and 6c). This finding corresponds to the separation of the 0.5–5 kbp fragments and can be attributed to the biased reptation mechanism. Because the 5 and 10 kbp fragments have approximately equal mobilities (*μ*_1_/*μ*_2_) at *E*_1_ and *E*_2_, they cannot be separated by the biased reptation mechanism. However, we obtained five flow streams in the frequency range from 0.5 to 10 Hz (Figures 5a and 6c) with >1 resolution between 2 and 10 kbp fragments. For the 5 and 10 kbp fragments, we expect that the reorientation mechanism contributes to the separation because the reorientation frequencies of these DNA lengths are 2 and 4 Hz, respectively. It was furthermore found that the calculated angles were lower than the measured angles. This difference can be explained by (1) the bent electric field lines and the variation in field magnitude throughout the microchip, which leads to larger than predicted angles (Supplementary Figure S1); and (2) the fact that the separated flow streams fluctuated along the *y* axis at low frequencies, leading to an increased uncertainty in the angle determination. A rough correction for the curved field lines can be based on the measured angle of the fluorescein sodium salt trajectory (Supplementary Video S5), for which *μ*_1_*/μ*_2_=1.

According to the field-dependent mobility mechanism, the best separation performance for all the DNA lengths is expected when *μ*_1_*/μ*_2_ varies maximally for all DNA lengths. According to Figure 6, the best separation performance should be achieved around *E*_2_≈25 V cm^−1^. In our observations, the highest resolution separation was indeed obtained within this field region, specifically when applying 59.5 V cm^−1^ (*E*_1_) and 22.4 V cm^−1^ (*E*_2_).

Figure 5c shows that five flow streams with good resolution were obtained when applying 134.6 V cm^−1^ (*E*_1_) at 10 Hz (the reorientation frequency of the DNA fragments in this field is 100 Hz for 0.5 kbp DNA and 5 Hz for 10 kbp DNA). At this frequency, both the field-dependent mobility (for small fragments) and switchback mechanisms (for large fragments) are expected to contribute to the resolution.

At low-frequency ranges (0.05–10 Hz) and an 89.8 V cm^−1^ *E*_1_, separation was consistently obtained for four flow streams, as seen in Figures 5d and 6b (6 Hz<*f*_or_=120 Hz for the DNA fragments). As another example, applying 29.1 V cm^−1^ (*E*_1_) at a 1 Hz frequency yielded a satisfactory separation (*R*_s,mean_≈0.7) with four independent flow streams (Figure 6d). At this field, 2 Hz<*f*_or_<40 Hz for the DNA fragments, indicating that the field-dependent mobility is the major contributor to the separation. As seen in Figure 6e, the switchback mechanism does not work well in the μGEL device when operated under low electric field strengths as the separation performance decreased when *f*_or_<0.1 Hz. The poorest separation was obtained when 9.7 V cm^−1^ (*E*_1_) was applied at the lowest frequency, 0.016 Hz. In this case, only two flow streams of fragments were separated.

The field-dependent mobility mechanism theoretically yields an angle *Φ* independent of the frequency. Figures 6a–e show this independence only at low frequencies. The observed decrease in angle with an increase in frequency is most likely due to the reorientation mechanism. For example, 5 and 10 kbp fragments were separated at 59.5 V cm^−1^ (*E*_1_) and 22.4 V cm^−1^ (*E*_2_) owing to the decreasing angle with increasing frequency. The agarose gel does not appear to yield satisfactory separations in the switching regime using the applied voltage protocol, despite the resolution decreasing for all field strengths at higher frequencies.

Throughout the experiments, the angle between *E*_1_ and *E*_2_ varied by ~90° (85–98°), which decreased to an angle of 60° at the exit location of the flow streams. In PFGE (where the reorientation mechanism is used), the best separation performances were always obtained when the angle between the two equal transverse electric fields was 120° (not 90°)^24^. However, in the case of the field-dependent mobility mechanism, the migration velocity differences determine the separation process. We optimally exploited the velocity differences with the applied orthogonal fields.

Up to 80 different experiments could be performed with the same microchip, using a large variety of voltage and frequency protocols, and yielding similar (>95%) results between the replicates of the experiments. Figure 6 shows the s.d. of the measurements performed using the same microchip. Different microchips could also be used for fractionation and purification with reproducible results (>70%).

For the DNA recovery calculations, we integrated the fluorescence over the width of the flow stream, both at the inlet and the outlet, and compared the results after subtracting the baseline intensity. For example, upon the alternating application of 59.5 V cm^−1^ (*E*_1_) and 24.6 V cm^−1^ (*E*_2_) fields at a 2 Hz frequency where the highest resolution was observed, the integrated fluorescence over the width of the flow stream at the inlet was 2897 (dimensionless number), and at the outlet (sum of all fragment streams) 2783 (dimensionless number). This corresponds to a recovery of 96.06%, which is as good as previously reported sequencing devices^29,30^.

The peak purity of the fragment streams was calculated using our resolution values by assuming Gaussian peak shapes. According to this analysis, the purity of the collected streams in Figure 5b are shown in Table 1.

Due to the low-height (20 μm) channels and separation chamber, Joule heating is not expected to contribute significantly to the separations in the applied field range. According to Supplementary Figure S4, Joule heating might have affected the separation performance at the highest field strengths (*E*_1_=134.6 V cm^−1^). The ionic strength of the buffer solution was kept constant throughout the experiments. The reservoir volume is 2000 times larger than the inner volume of the microchip and the buffer solution was frequently refreshed.

The μGEL device has a calculated throughput of 0.18 ng of molecules per hour at the DNA input concentration of 12.5 ng μL^−1^ and is comparable to previously reported microfabricated devices^20,31,32^.

In our experiments, we found that the width of the band at the injection site (110 μm) did not significantly differ from the width of the bands at the flow stream exit locations (120–200 μm). Thus, band broadening was minimal during the separation process, which is in accordance with the results suggested by Huang *et al.*^12^ and Lerch *et al.*^33^ At low frequencies, however, the sawtooth movement of the DNA fragments is a major contributor to band broadening. The contribution of the alternating field protocol can be quantified by

(2)Lfr=0.5µ2E2f(tanθ1cosθ2+sinθ2)

where *L*_fr_ is the distance along the vertical axis travelled by a DNA fragment in one switching period. According to Equation (2), the contribution of the field switching (that is, fluctuation of fragment flow streams along the separation matrix) to the total band broadening will be less than 30% (~33 μm band width) when *f*<0.05 Hz and *E*_2_=24.6 V cm^−1^. Because the width of the injection band is the main contributor to the band variance, the resolution of the μGEL device could be improved by decreasing the injection channel width.

The peak width variances between the injection channel and flow stream exit locations at >10 Hz and >2 Hz are <5% and 15%, respectively. Peak width variations are given in Figure 5.

For other ionic species, no reptation occurs and the molecules have the same mobility under both electric fields. Thus, the *2fL* term drops and *μ*_2_*=μ*_1_, reducing Equation (1) to

(3)Φ=atan(E1sinθ1+E2sinθ2E1cosθ1+E2cosθ2)

Periodic application of *E*_1_ and *E*_2_ results in the ionic species following a path at an angle *Φ*_0_ > *Φ* (Supplementary Video S5)^27^. When the applied field strengths were 59.5 V cm^−1^ (*E*_1_) and 22.4 V cm^−1^ (*E*_2_), we calculated *Φ*_0_=45° for the fluorescein sodium salt, predicting clear separation from the DNA fragments with a calculated *Φ*<38.5° (Supplementary Figure S2). We experimentally confirmed the purification by separating a mixture of 0.5–10 kbp fragments and 10 mM fluorescein sodium salt by applying 59.5 V cm^−1^ (*E*_1_) and 22.4 V cm^−1^ (*E*_2_) at 2 Hz (Supplementary Video S5).

The advantages of the proposed device include its simple design and fabrication process. Here we used the device to separate sub-10 kbp DNA fragments. However, the use of agarose gel as the sieving matrix provides flexibility, which in principle enables fractionation of DNA fragment sizes outside the 0.5–10 kbp range studied here by changing the gel concentration. A similar strategy is used in the commercially available mate-pair sequencing device, BluePippin (SageElf Inc.), which performs capillary electrophoresis for DNA fractionation in a precast agarose gel cassette with orthogonal sample plug extraction wells^34^. BluePippin can process between a few tens to thousands of base pairs, simply by changing the agarose concentration and voltage protocol. However, fractionation of sub-10 kbp DNA fragments requires 4 h using BluePippin^9^, while the μGEL device presented here requires only 2 min.

The band resolution for 0.5 to 10 kbp fragments and fluorescein sodium salt varied between 0.9 and 1.4, when 59.5 and 24.6 V cm^−1^ fields were switched at a frequency of 2 Hz. Because the main contributor to the band variance was the injection band width, we expect that resolution in the μGEL device can be further improved by decreasing the width of the injected band. Improvements in the band separation will be attempted by optimizing the voltage protocol to separate the fragment sizes. Chen *et al.*^23^ predicted that large and small fragments at low frequencies (<2*/t*_or_) will migrate through isotropic matrices such as agarose gels at similar angles and no continuous flow fractionation would be possible. However, *μ*_1_/*μ*_2_ was assumed to be constant by Chen *et al.*, which is incorrect. As a result, we could achieve fractionation using the field-dependent mobility of DNA.

## Conclusions

We have introduced a novel microfluidic platform for the purification and high-resolution fractionation of DNA molecules. A new separation principle is explained based on variations in the field-dependent mobility of DNA molecules with respect to their length. The use of the agarose separation matrix enabled simple, fast, and extremely low-cost fabrication. This device would be of a broad interest for second-generation sequencing and clinical diagnosis applications, as it can achieve similar performance with much less effort in terms of fabrication and operation in comparison with currently available devices. In addition, the μGEL device can further be extended to protein gel electrophoresis by replacing the agarose gel with a polyacrylamide gel^35^.

## Figures and Tables

**Table 1:** Peak purity of the collected streams in Figure 5b

Electric field (V cm^−1^)		Fragments	*R*_s_	Percentage peak overlap (%)	Peak purity (%)
*E*_1_	*E*_2_				
59.5	24.6	Between 0.5 and 1 kbp	1.3	0.50	99.5
		Between 1 and 2 kbp	0.6	11.50	88.5*
		Between 2 and 5 kbp	1.0	2.30	97.7
		Between 5 and 10 kbp	1.4	0.25	99.7
