# Supplementary material for: Exploiting biased reptation for continuous flow preparative DNA fractionation in a versatile microfluidic platform
Source: Microsyst Nanoeng. 2017 May 22;3:17001. doi: 10.1038/micronano.2017.1 (PMC6444976; doi:10.1038/micronano.2017.1)
Supplement: Supplementary Information [file micronano20171-s1.pdf]

Supplementary file

Exploiting biased reptation for continuous flow preparative DNA fractionation in a versatile microfluidic platform

Burcu Gumuscu, Johan G. Bomer, Hans L. de Boer, Albert van den Berg and Jan C. T. Eijkel

Microsystems & Nanoengineering (2017) **3,** 17001; doi:10.1038/micronano.2017.1; Published online: 22 May 2017

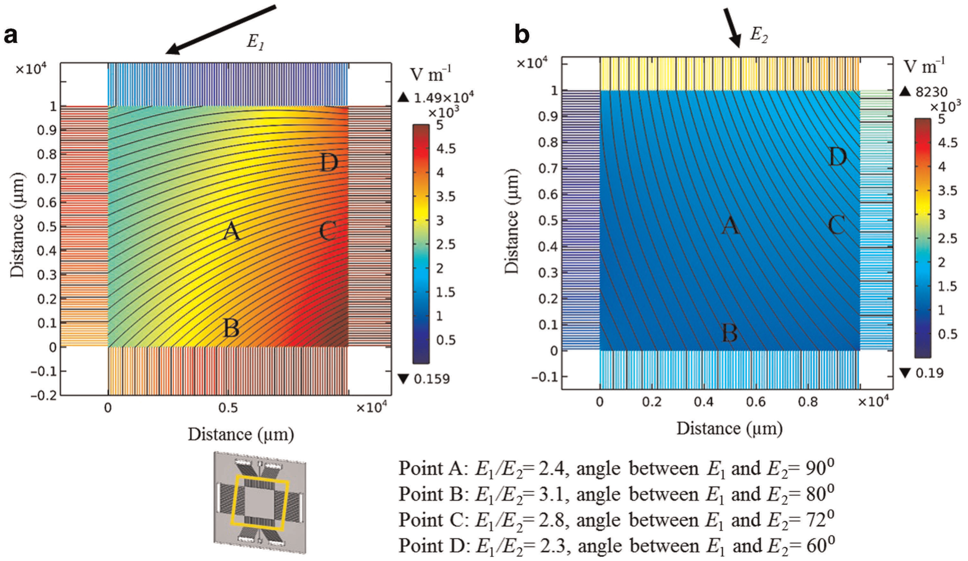

Figure S1 Simulated electric field distribution in the microchip. (**a**) Electric field profile during the application of E_1 and (**b**) electric field profile during the application of E_2. Injection microchannel is placed at the bottom in the middle of the separation matrix. The black lines present the field direction and the colors present the field strength.

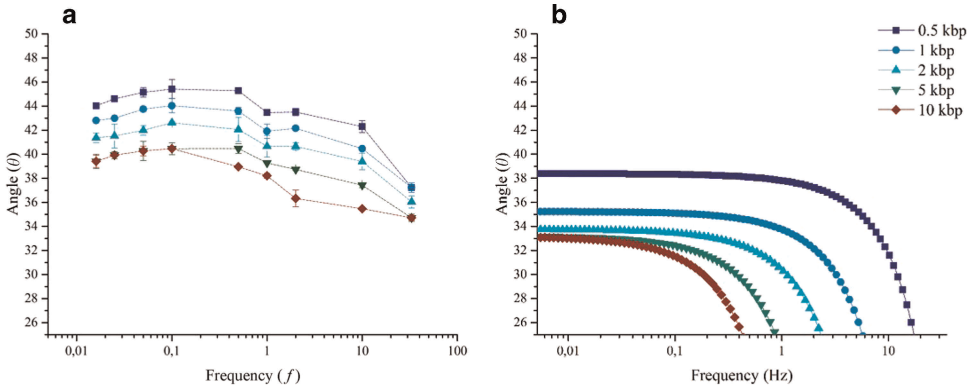

Figure S2 Deflection angle θ variation for individual DNA fragments at different frequencies. (**a**) Measured deflection angles at $E_1 = 59.5$ V cm^{-1} and $E_2 = 24.6$ V cm^{-1}. (**b**) Calculated deflection angles for the same fields.

BIOS Lab-on-a-Chip Group, MESA+ Institute for Nanotechnology, MIRA Institute for Biomedical Technology and Technical Medicine, University of Twente, 7500 AE Enschede, The Netherlands
Correspondence: Burcu Gumuscu (b.gumuscu@berkeley.edu) or Jan C. T. Eijkel (j.c.t.eijkel@utwente.nl)

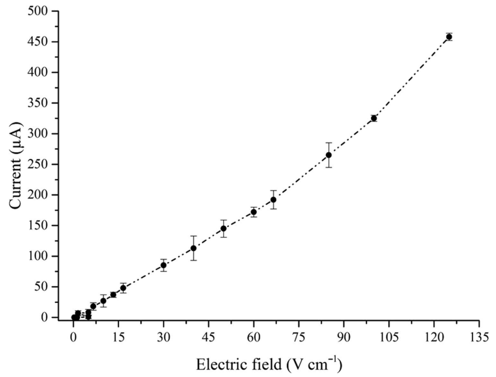

Figure S3 Current-voltage graph demonstrating the absence of Joule heating in the microchip at the applied field strengths.

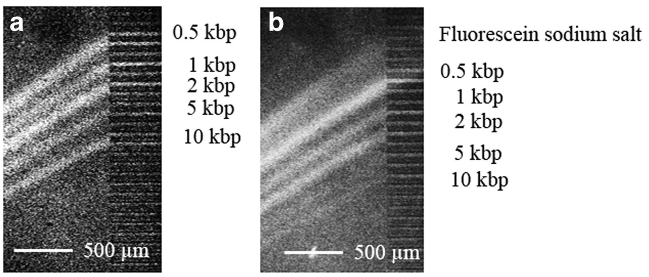

Figure S4 DNA purification from the fluorescein sodium salt in a continuous flow. Fluorescence images present separation of (**a**) only 0.5–10 kbp DNA fragments and (**b**) of 0.5–10 kbp DNA fragments and fluorescein sodium salt when $E_1 = 59.5\ \mathrm{V\ cm^{-1}}$ and $E_2 = 24.6\ \mathrm{V\ cm^{-1}}$ were applied at a 2 Hz frequency. Each image was recorded with a 12 s exposure time.

Supplementary Video S5. Video of large DNA fragments (10 kbp) under $59.5\ \mathrm{V\ cm^{-1}}$ (E_1) and $24.6\ \mathrm{V\ cm^{-1}}$ (E_2) electric fields at a 0.016 Hz frequency. Agarose concentration is 1.2%, exposure time is 0.15 s, and the precision of the orientation time is ± 0.15 s.
